# Beyond unit-level certification: An operational agenda for biodiversity-positive tropical production landscapes

**DOI:** 10.1016/j.isci.2026.117444

**Published:** 2026-09-15

**Authors:** Joeri A. Zwerts

**Affiliations:** 1 Wildlife Ecology and Nature Restoration, Utrecht University, Padualaan 8, Utrecht 3584CH, the Netherlands

**Keywords:** jurisdictional approaches, voluntary sustainability standards, VSS, biodiversity monitoring, blended finance, biodiversity credits, agro-commodities

## Abstract

Rising global demand for tropical agro-commodities such as palm oil, cocoa, timber, and soy, intensifies pressure on the forests that hold most terrestrial biodiversity. Unit-level sustainability certification has largely failed to halt biodiversity loss, constrained by three interacting barriers: translating species’ persistence requirements into practice, an annual biodiversity financing shortfall near US$711 billion, and fragmented governance. In this perspective, I argue for a shift from certified management units to biodiversity-positive production landscapes, structured around three interdependent domains: ecological requirements and outcome verification, sustainable finance, and integrated governance. Automated sensing can now yield field-validated biological key performance indicators (bio-KPIs) at landscape scale; tying these outcome metrics to financial instruments like concessional debt, first-loss structures, and biodiversity credits, reframes finance as an ongoing return on measured performance. Jurisdictional traceability renders territory-wide claims auditable and aligns them with the Kunming-Montreal Global Biodiversity Framework. Together, these domains offer a route to landscapes that sustain both commodity production and biodiversity.

## Graphical abstract

**Figure:**
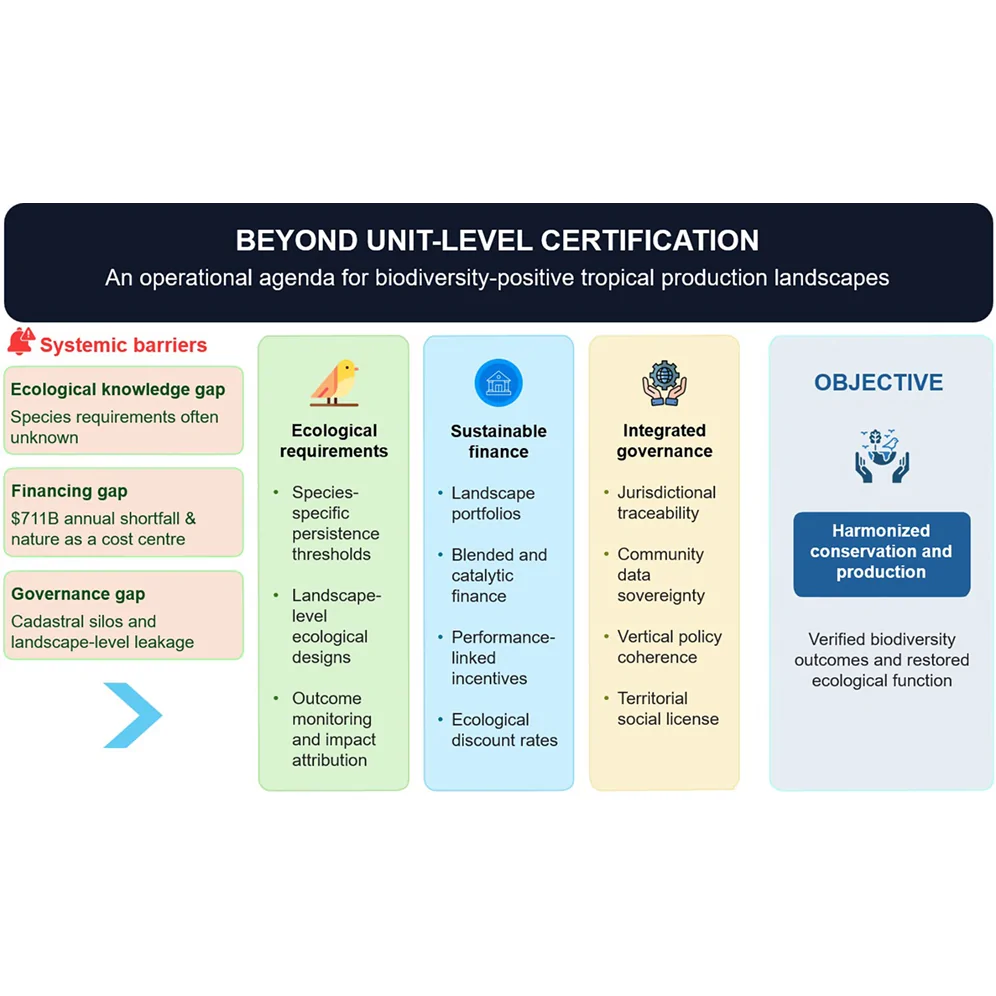

## Teaser

Environmental science; Environmental policy; Nature conservation

## Introduction

The tropics are the stage for a defining challenge of the Anthropocene: reconciling the necessity of agro-commodity production, encompassing food, fiber, and timber, and the imperative of biodiversity preservation.^1^ While tropical forests harbor 62% of all described terrestrial vertebrate species,^2^ they face accelerating degradation and loss driven by the expansion of agro-commodity production to meet global consumption patterns.^3^

Pressures on biodiversity vary by region. In Southeast Asia, oil palm remains the dominant driver of land-use change, creating fragmented mosaics of monoculture and remnant forest patches.^4^^,^^5^ In the Amazon, the cattle-soy complex drives frontier expansion, converting rainforest into pastures and arable land.^6^ The West and Central African cocoa belt encroaches upon remaining forest reserves,^7^ and across the pan-tropical zone the road networks on which selective logging depends fragment forests, elevate fire and erosion risk, and open remote interiors to hunters.^8^ As the global population nears 8.5 billion and welfare increases, a paradox intensifies: humanity must extract more from finite land without collapsing the ecological systems that sustain that very production.^9^^,^^10^

Resolving this paradox requires recognizing that biodiversity is the biological machinery of these production landscapes. It is not merely a collection of species to be spared from production in isolated reserves, but the source of essential ecosystem services, including pollination, natural pest control, seed dispersal, and soil nutrient cycling, that provide the resilience and insurance policy required to maintain agro-commodity yields in an increasingly volatile climate.^11^ Biodiversity matters because of response diversity: the variation among species in how they react to environmental change. Multiple species performing similar ecological roles offer little insurance if they all respond to a disturbance in the same way; what sustains a function under shifting conditions is the spread of responses within the group that delivers it.^12^ When fragmentation and degradation narrow that range of responses, the system loses its capacity to regenerate aftershocks like fire or drought, undermining the long-term viability of the agricultural matrix itself.

### The case for a landscape lens

For the past two decades, the primary mechanism for mitigating the impact of agro-commodities on biodiversity has been through voluntary sustainability standards (VSS) such as the Roundtable on Sustainable Palm Oil (RSPO), the Forest Stewardship Council (FSC), and the Round Table on Responsible Soy (RTRS). The underlying premise is that if individual management units adhere to strict principles, such as high conservation value (HCV) assessments and zero-deforestation commitments, biodiversity will be conserved. However, the efficacy of this approach is a subject of intense scientific debate.^13^^,^^14^

Direct evidence that certification itself delivers biodiversity outcomes remains sparse. A clear case comes from the Congo Basin, where FSC-certified logging concessions recorded significantly higher encounter rates of large mammals, including critically endangered gorillas and forest elephants, than non-certified concessions.^15^ This suggests that timber production landscapes are not necessarily biological deserts, provided specific management interventions such as enforcement of hunting bans are in place. Yet a landscape that retains large mammals can still be an ecological shadow of untouched forests. Across birds, plants, dung beetles, orchid bees, and ants in the Brazilian Amazon, biotic homogenization was most pronounced in production areas, where disturbance-tolerant, well-dispersing species progressively displaced specialists.^16^

Moreover, the connectivity of management units is often not assessed or guaranteed by a certification standard. In Peninsular Malaysia, RSPO-certified and uncertified large-scale plantations proved equally impoverished in terms of landscape heterogeneity, while uncertified smallholdings were many times more heterogeneous.^17^ If a certified plantation protects a patch of forest, but that patch is completely isolated by a sea of monoculture and hostile land uses, the conservation benefit is severely capped.^18^ The patch becomes a sink or a genetic island, potentially leading to a delayed biological collapse as species linger in a fragmented habitat for generations, subject to physiological and generational stressors such as herbicide exposure, replanting or harvesting cycles, and chronic disturbance, before disappearing due to inbreeding and stochastic events.^19^^,^^20^

This highlights a fundamental flaw in the management unit-level approach: ecological boundaries rarely align with cadastral boundaries. A jaguar, an elephant, or a hornbill does not recognize the border between a certified unit and a non-compliant neighbor. Without landscape-level oversight, even well-managed units and set-asides risk becoming paper parks—areas that are protected on maps and in management plans but suffer from degradation, due to a lack of connectivity.^21^

### From silos to synthesis

Many ecological, social, and economic challenges cannot be solved within a single management unit, necessitating a shift toward landscape approaches.^18^ No single landscape approach transfers unchanged across contexts; rather, each landscape requires context-specific strategies adapted to local ecological conditions, land tenure, commodity sectors, governance capacity, and social priorities. Nevertheless, many production frontiers face recurring landscape-scale challenges: ecological connectivity requires collaboration across multiple management units; anti-poaching requires patrols and intelligence sharing that transcend property lines^22^; and watershed management is inherently spatial, as upstream land users affect downstream buffers. Finally, landscape approaches must address leakage, where protection or intensification in one area displaces land-use pressure elsewhere, including through indirect land-use change^23^, as ecological and social goals are not automatically synergistic.

This perspective builds on a substantial body of work on landscape approaches, which have long emphasized connecting ecological integrity, planning, and governance across spatial scales.^18^^,^^24^^,^^25^ Most closely related, Morgan et al.^26^ proposed a framework for integrated forest landscape management, arguing that sustainable production, ecosystem conservation and restoration, and inclusive governance must be pursued simultaneously to achieve landscape-scale sustainability. Here, I focus on a narrower operational challenge specific to tropical agro-commodity frontiers: *how* production and conservation can move beyond unit-level certification toward more integrated, landscape-scale, biodiversity-positive stewardship. Like Morgan et al., I draw on ecological and governance dimensions, but add performance-linked finance. Performance-linked finance already reaches nature,^27^^,^^28^^,^^29^ yet where it targets biodiversity, it triggers on area- or practice-based proxies such as hectares restored, share of certified sourcing. Such triggers can be met while a landscape is ecologically hollowed out, and the rare instruments that do pay on biological outcomes remain single-species and site-bound. I argue that these existing mechanisms, paired with the automated, landscape-scale, multi-taxon verification now becoming feasible, can tie the cost of the capital already flowing through production landscapes to what actually lives in them.

To address this, I discuss three interdependent operational dimensions required to move from unit-level certification toward biodiversity-positive production landscapes: (1) ecological requirements and outcome verification, (2) sustainable finance, and (3) integrated governance. Together, these dimensions provide the scientific, financial, and institutional foundations required to operationalize sustainable agro-commodity landscapes.

## Ecological requirements and outcome verification

Effective landscape approaches must be grounded in a robust ecological understanding of how biodiversity responds to human-modified landscapes. These landscapes consist of mosaics of remnant natural habitat embedded within matrices of varying permeability, including agroforestry systems, monocultures, and pasture. Despite their ubiquity, a fundamental ecological knowledge gap persists. Species survival and population persistence in human-modified landscapes remain poorly understood, particularly with respect to species-specific ecological thresholds.^30^^,^^31^^,^^32^^,^^33^ As a result, management interventions often rely on coarse or generic assumptions, overlooking extinction debts (delayed local extinctions still owed as a result of past habitat loss) that accumulate as populations persist in sub-optimal fragments.^19^

### Species-specific ecological requirements: Extent, connectivity, and quality

Designing landscapes that sustain biodiversity requires moving beyond one-size-fits-all thresholds toward species-specific ecological requirements. Species persistence in human-modified landscapes is governed by three linked requirements: habitat extent, functional connectivity, and habitat quality. These requirements are not properties of forest patches alone, but are also shaped by the structure, composition, and disturbance regimes of the surrounding production matrix.

Habitat extent defines the minimum area required to sustain a viable population. Although broad landscape-level benchmarks—such as retaining 40% forest cover, with ∼10% in a single large patch and the remaining 30% distributed among smaller patches embedded within a high-quality matrix—provide useful starting points,^34^ spatial requirements vary substantially among taxa. Large carnivores and wide-ranging megafauna may require thousands of hectares, whereas smaller mammals, birds, amphibians, or insects can persist in much smaller fragments, provided these are not fully isolated and are embedded within a sufficiently permeable matrix.^33^

Functional connectivity captures the landscape’s effective permeability to movement and gene flow and is a key determinant of population persistence in fragmented systems.^35^ Unlike structural connectivity, which reflects the physical configuration of habitat patches, functional connectivity depends on how species perceive and navigate the matrix. Corridors that appear connected on maps may be unusable if they are too narrow, too exposed, too disturbed, or thermally unsuitable. Dispersal kernels, describing the probability of movement over distance, vary markedly among taxa, producing highly contextual barriers to connectivity.^36^^,^^37^ For example, a dirt road may facilitate movement for a wide-ranging jaguar while constituting a near-absolute barrier for a forest-interior bird.

Finally, habitat quality determines whether remnant patches and surrounding matrix elements remain ecologically functional. High-quality habitats must provide critical resources such as nesting sites, refuges, and temporally stable food supplies, while maintaining suitable microclimatic conditions and limiting edge effects such as wind exposure, increased light penetration,^38^ elevated predation and hunting.

In production landscapes, connectivity and habitat quality are strongly shaped by matrix heterogeneity: mosaics that include forest fragments, riparian buffers, wetlands, agroforestry elements, smallholder plots, mixed-age stands, or structurally diverse understorey vegetation can provide more resources, refuges, and movement opportunities than simplified monocultures. This does not imply equivalence with natural forest, but it does mean that certification and landscape planning should assess not only set-asides, but also how matrix heterogeneity, permeability, and disturbance regimes affect species persistence. Failure to account for these fine-scale and landscape-scale conditions risks creating fragments that remain structurally intact but lack the resources, connectivity, or demographic conditions required to sustain viable populations across generations.

### Sensor innovations and automated monitoring

Impact cannot be managed if it cannot be measured. Rigorous monitoring has historically been hindered by high costs and scaling challenges.^39^^,^^40^ Although satellite remote sensing has established deforestation tracking as a standard benchmark for financial and governance frameworks^41^^,^^42^; it cannot detect the empty forest syndrome.^43^ This occurs when landscapes appear structurally intact from above but have suffered internal ecological collapse due to hunting, invasive species, or climate stress.^44^ Consequently, sustainability cannot be verified from above alone: it requires state-of-the-art, yet readily available, measurements of biological outcomes from beneath the canopy.^45^^,^^46^^,^^47^

The conservation sector is witnessing a rapid expansion of technological capabilities to address this.^48^ Acoustic multispecies detection models now leverage deep learning to identify individual taxa,^49^ while computer vision and on-board edge processing enable camera traps to provide real-time alerts for high-priority species via satellite link.^50^ These AI-driven advancements allow for the rapid classification of millions of audio files and images, yielding the high-resolution biological datasets necessary for verified landscape-scale management, hereafter referred to as biological key performance indicators (bio-KPIs).^51^^,^^52^ Moreover, bioacoustic indices allow for the tracking of broad community dynamics,^53^^,^^54^^,^^55^ while environmental DNA (eDNA) provides an efficient snapshot of species presence.^56^ The use of drones is revolutionizing aerial monitoring and thermal sensors represent a breakthrough in monitoring canopy-dwelling wildlife, enabling population counts of arboreal species once considered nearly impossible to survey.^57^^,^^58^ Simultaneously, LiDAR technology provides the ability to map the intricate three-dimensional architecture of forest canopies in great detail. Together, these technologies can provide a comprehensive snapshot of landscape-wide biodiversity that allows for data-driven management at a scale previously unimagined.

### Rigorous experimental designs for impact attribution

A primary challenge in applying biomonitoring techniques is the necessity for rigorous, standardized experimental designs to link observed changes to management. Establishing clear attribution of outcomes requires standardized monitoring characterized by robust frameworks, such as before-after-control-impact (BACI) designs.^59^ Such designs directly address the knowledge gap concerning the long-term effectiveness of conservation measures. This rigor is not merely academic: without it, biological change cannot be separated from background variation, and conservation outcomes cannot be verified—or rewarded.

### Data sovereignty and community ecology

The deployment of sensor grids in tropical forest landscapes introduces ethical challenges regarding data ownership and sovereignty.^60^ Without careful consideration, monitoring risks is becoming a form of digital colonialism, where ecological data are extracted by external actors without the consent or benefit of local communities. A model of community-tech integration, where data are transparent and co-managed with local and indigenous groups, ensures that local populations act as landscape guardians rather than passive subjects of surveillance. This extends to research leadership itself: monitoring programmes should build local scientific capacity and institutional partnership, not simply local data access, to avoid the extractive dynamics of externally led research. By flipping the narrative toward bottom-up empowerment, technology becomes a tool for local sovereignty and successful long-term landscape stewardship.

## Sustainable finance

Biodiversity conservation has traditionally been framed as a cost center or a philanthropic endeavor, rather than a productive investment in natural capital. This has led to a staggering global biodiversity financing gap, estimated at approximately US$711 billion per year.^61^ This underpins the degradation of commodity frontiers, as structural economic incentives favor total conversion over costly conservation set-asides. Current international funding remains grossly inadequate to meet the scale of the crisis, necessitating a fundamental rethink of how landscape-scale conservation is valued and financed. Sustainable finance, mechanisms that integrate environmental, social, and governance (ESG) criteria into financial decisions to generate long-term competitive financial returns and positive societal impact, has the potential to drive management improvements at landscape scale. By moving stewardship beyond the internal boundaries of a single management unit, practitioners can address *trans*-boundary issues, such as functional connectivity and watershed health that are impossible to resolve through fragmented unit-level certification alone.

### Ecological discount rates

A landscape approach requires a shift in how natural capital is valued over time. Conventional financial decision-making often applies high private discount rates, causing future ecological benefits to be heavily devalued relative to immediate extraction returns. In such frameworks, biodiversity outcomes decades into the future can appear economically negligible compared with short-term revenues. A lower, ecologically informed discount rate—one that gives greater weight to future biodiversity benefits and the long-term consequences of ecosystem change—keeps future conservation outcomes closer to their present value and can improve the economic case for long-term conservation.^62^^,^^63^

The primary barrier to implementing such a rate is the prevailing fiduciary short-termism in capital markets, where investment incentives and performance metrics often prioritize short-term financial returns over long-term ecological and systemic risks. Addressing this requires either broadening fiduciary mandates to encompass long-term well-being and environmental stewardship, and/or deploying catalytic capital structures that absorb long-term risks and thereby enable private investment over generational time horizons. This adjustment recognizes that delayed investment in ecological integrity allows environmental liabilities—such as extinction debt and the erosion of ecosystem functions—to accumulate over time.^20^ Connectivity and restoration investments can therefore be framed not as philanthropic expenditures, but as long-term risk mitigation strategies that protect the natural capital underpinning future economic stability.^64^

### The value chain vs. the landscape portfolio

Historically, investment has been structured vertically along commodity value chains, focusing on individual corporate entities to capture the economic flow of a single product. Sustainable production landscapes instead require landscape portfolios that shift the investment focus from isolated assets toward entire territories. The obstacle is ticket size: institutional investors such as pension funds and insurers manage trillions in assets, but cannot efficiently deploy capital into the small deal sizes typical of tropical forest landscapes. Landscape portfolios address this by bundling fragmented agro-commodity producers into scalable, investible entities—which in turn requires a layer of intermediary organization, such as landscape coordination bodies or regional cooperatives that can standardize data, manage territorial risks, and act as a single point of entry for institutional capital.

Once production entities are created, portfolios can expand to include conservation and restoration initiatives, integrating ecological and productive assets into a single diversified, territory-wide asset class. This bridges the structural gap between global capital and ground-level ecological realities, financing conservation as part of a functional economic landscape.^65^ Bundled portfolios can also mitigate idiosyncratic risks—unit-specific failures such as localized weather events or pest outbreaks—while capturing landscape-wide synergies in ecosystem service provision. Yet territory-wide bundling remains rare: coordinating across fragmented land titles is costly, and the intermediary layer that would absorb that cost seldom exists.

### Blended finance

A central challenge in financing these portfolios is the perceived high risk of investing in tropical agro-commodity frontiers, which encompasses both large-scale industrial concessions and smallholder-dominated mosaics. Blended finance serves as a catalytic tool by using public or philanthropic funds to de-risk and mobilize private investment.^66^^,^^67^ This is achieved through risk-sharing mechanisms such as first-loss equity—where public funds absorb initial losses—or concessional debt, which provides loans with below-market rates and extended grace periods. These instruments are also important for smallholders who are often excluded from traditional credit markets due to a lack of recognized land titles or capital assets.^68^ Moreover, active impact investing, rather than divestment, is what drives change: research suggests simple divestment from bad actors rarely shifts the cost of capital enough to alter corporate behavior.^69^

These instruments can be further optimized through smart contracts—self-executing blockchain agreements that automate financial incentives based on verified ecological performance.^70^ Linked to real-time monitoring feeds (see ecological requirements and outcome verification), these contracts can trigger interest rate reductions or principal forgiveness when landscape-level targets are verified, reducing administrative overhead and ensuring immediate rewards for conservation success.^71^ This transforms conservation from a secondary obligation into a primary driver of financial performance, directly tying loan terms to ecological outcomes.

### Biodiversity credits and supply chain security

Biodiversity credits can be a primary financial instrument to complement landscape strategies directly financing the restoration and protection initiatives essential to ecosystem health.^61^^,^^72^ They function by quantifying ecological improvements—such as species richness, habitat connectivity, or ecosystem integrity—into measurable, tradeable units. Unlike fungible carbon credits, biodiversity is inherently local; a credit generated in the Amazon cannot offset destruction in Borneo. Biodiversity risk is also inherently local, as the accumulation of extinction debt represents a long-term financial liability. For a commodity buyer, this debt is a hidden systemic risk: the ecosystem services they depend on today can already be scheduled for collapse unless restoration is funded.^73^ Commodifying outcomes through credits allow producers to internalize the value of their conservation efforts, turning a historical compliance cost into a revenue stream for stewardship.

To protocolize this finance-to-ecology link, we must move beyond simple forest cover as the dominant biomonitoring metric,^41^ whereby more nuanced bio-KPIs are incorporated to serve as triggers for financial instruments. For example, functional connectivity scores derived from field-validated movement models,^37^ could measure the permeability of the agricultural matrix. By embedding these high-resolution ecological metrics into landscape-linked loans, we ensure that financial incentives are aligned with the actual functional recovery of the ecosystem.

## Integrated governance

Most environmental mitigation operates within cadastral silos—individual management units—despite the *trans*-boundary nature of ecosystems. This governance gap prevents alignment between local land-use planning and global supply chains. Without a jurisdictional scaffold to bridge these silos, individual units remain ecologically vulnerable, and capital investments in landscape portfolios remain precarious.

### Jurisdictional traceability

Moving from isolated units to cohesive landscape governance requires jurisdictional traceability, in which entire administrative territories (e.g., districts or provinces) are audited against shared sustainability targets established by multi-stakeholder coalitions.^25^^,^^74^ Traditionally, traceability is a vertical exercise, tracking a product from a specific farm to the consumer. In contrast, jurisdictional traceability is a horizontal exercise that verifies the integrity of an entire landscape, for which innovative monitoring (sensor innovations and automated monitoring) is the technological backbone for regional claims; by aggregating high-resolution ecological data governments can provide verified proof of landscape integrity to international buyers. Within this architecture, the state must transition from a distant regulator to an active convener.

This mechanism is transformative for three reasons. First, it can partly shift the compliance and verification cost from the individual producer to the jurisdictional infrastructure, which is particularly vital for smallholders who often lack the capital for individual certification. Second, it allows global buyers to classify entire regions as low-risk, streamlining due diligence. Third, it creates a collective responsibility: if the biological health of the landscape declines, the entire jurisdiction is at risk. These data-driven transparency provides the essential legal scaffold upon which the ecological and financial dimensions are constructed.

### Social equity

Much like the environmental issues discussed in previous chapters, social challenges in tropical frontiers are rarely contained within a single management unit. Land tenure conflicts, resource competition, labor migration, and human-wildlife conflict often span multiple concessions and community lands, creating systemic risks that a single operator cannot mitigate in isolation.^75^ Traditionally, the social license to operate (SLO)—the approval of an operation by local stakeholders—has been viewed as a site-specific agreement at the level of a single management unit. This is distinct from free, prior, and informed consent (FPIC), the right of communities to grant or withhold agreement to projects affecting their lands: SLO is reputational and judged from the operator’s side, whereas FPIC is a right established for indigenous peoples in the United Nations Declaration on the Rights of Indigenous Peoples, and extended to local communities under both RSPO and FSC principles and criteria.

However, just as we should move toward landscape-scale ecological monitoring, we must move from site-specific agreements to a collective territorial contract. In this model, local communities and indigenous groups grant legitimacy not to an individual company, but to the broader production landscape as a functional whole.

The necessity of a territorial approach stems from the fact that localized social standards can result in social leakage, where conflict or land-tenure pressure is simply displaced onto neighboring, unmanaged community lands. By transitioning to a landscape-scale framework, stakeholders can harmonize the often-disparate interests of large-scale corporate concessions and smallholder plots, creating shared infrastructure and grievance mechanisms that reduce the antagonistic dynamics typical of site-specific models. Ultimately, because individual companies are often transient while the landscape remains, anchoring equity in a territorial contract built from community-level FPIC ensures that community rights are tied to enduring regional governance rather than the fluctuating corporate social responsibility budgets of a single firm.

### Global policy integration: EUDR, KMGBF, and OECMs

This vision of integrated jurisdictional governance aligns directly with the trajectory of global policy, specifically the EU Deforestation Regulation (EUDR) and the Kunming-Montreal Global Biodiversity Framework (KMGBF). The success of landscape governance depends on vertical policy coherence, whereby local land-use planning and zoning are explicitly aligned with international supply chain requirements such as the EUDR. In principle, this allows local legal commitments—such as protecting ecological corridors—to be reinforced by market incentives. In practice, however, these incentives have largely failed to materialize. Indonesia’s jurisdictional initiatives—the most advanced test case—illustrate this chicken-and-egg problem: jurisdictions hesitate to commit without market rewards, while buyers hesitate to reward without demonstrated performance.^76^ Breaking this deadlock require credible, comparable evidence of jurisdictional performance, exactly what the ecological requirements and outcome verification dimension is designed to provide.

The KMGBF provides the global political mandate for landscape-scale interventions by establishing clear, multi-lateral targets that production frontiers must help meet. Well-managed production landscapes are increasingly recognized as candidates for designation as other effective area-based conservation measures (OECMs), which already contribute measurably, if still marginally, to global target 3 (30 × 30) coverage.^77^ Realizing that potential for production landscapes specifically will again depend on demonstrating the same sustained, verified biodiversity outcomes.

Furthermore, jurisdictional governance addresses the requirements of target 15, which mandates that large and transnational companies and financial institutions regularly monitor, assess, and transparently disclose their risks, dependencies, and impacts on biodiversity. By providing verified, territory-wide data, the jurisdictional approach simplifies corporate compliance with the Taskforce on Nature-related Financial Disclosures.^42^ Finally, by aligning with Target 19, which aims to mobilize $200 billion per year for biodiversity, this framework creates a powerful sovereign incentive for nations to support sustainable landscape initiatives not merely as environmental projects, but as central components of their National Biodiversity Strategies and Action Plans (NBSAPs).

## From framework to practice

**Figure 1:**
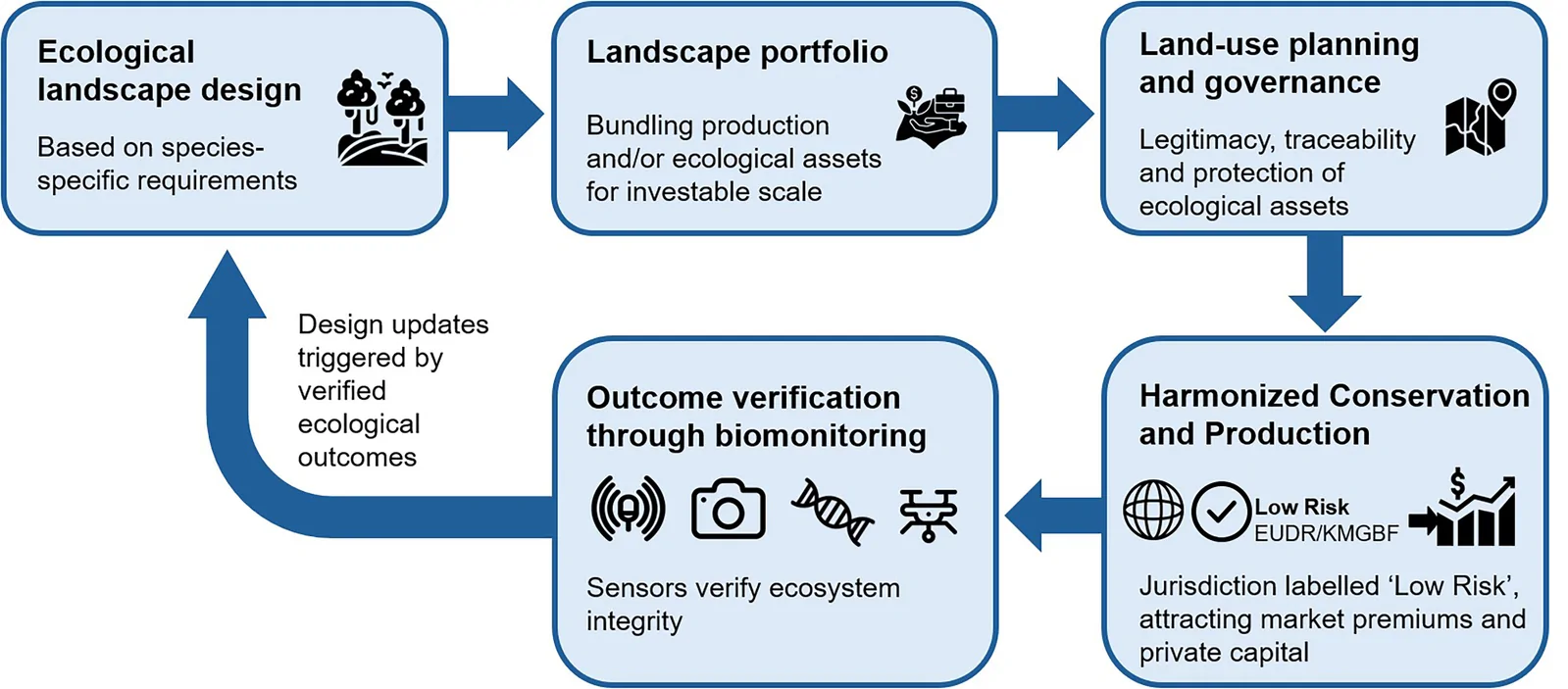
The operational cycle for biodiversity-positive production landscapes This schematic illustrates the operational feedback loop between ecological landscape design, landscape finance, governance, and outcome verification.

First, a landscape’s spatial boundary must be defined pragmatically, combining ecological criteria such as watersheds or species ranges with existing administrative or jurisdictional units, rather than following a single fixed rule.^25^ To translate this framework into practice, I propose a four-stage operational roadmap: (1) ecological landscape design and baseline characterization to establish field-validated bio-KPIs; (2) the structuring of a landscape portfolio and performance-linked financial instruments tied directly to these metrics; (3) writing jurisdictional governance commitments into binding zoning law and policy; and (4) continuous iterative monitoring to trigger financial reward or mitigation pathways (Figure 1).

The first stage, ecological design and baseline establishment, is best carried out by conservation research institutions or specialist ecological monitoring actors, using sensor grids to establish field-validated bio-KPIs (sensor innovations and automated monitoring)—a commissioned, ongoing monitoring role rather than one-off, grant-dependent fieldwork. Standardized protocols are essential here, as trend attribution depends on consistent monitoring methods over time (rigorous experimental designs for impact attribution), carried through by the same actors into stage 4. The second stage, structuring performance-linked financial instruments, falls to impact investors, development finance institutions, and blended finance vehicle managers, often anchored by commitments from downstream commodity buyers—bundling production and conservation land into landscape portfolios (the value chain vs. the landscape portfolio), de-risked through blended finance (blended finance), and disbursed via smart contracts or biodiversity credits calibrated to the stage 1 baseline (biodiversity credits and supply chain security). This offers investors a de-risked, landscape-scale asset class with long-term returns (ecological discount rates) and offers commodity buyers verified, regulation-compliant supply security in return (biodiversity credits and supply chain security). The third stage, writing governance commitments into binding zoning law and policy, is led by jurisdictional governments acting as convener (jurisdictional traceability), in coalition with producer associations and communities engaged through territorial contracting (social equity). This benefits governments through streamlined buyer due diligence and enhanced market access for the whole jurisdiction (jurisdictional traceability), and benefits smallholders and communities by shifting individual certification costs onto shared jurisdictional infrastructure while securing recognition of land rights (social equity). In practice, stages 2 and 3 are often pursued iteratively rather than strictly sequentially: financial commitments can create the incentive for jurisdictions to formalize governance frameworks, just as governance certainty can unlock financing that would otherwise be considered too risky. The fourth stage, continuous iterative monitoring, requires independent verifiers to audit ongoing ecological data against the stage 1 baseline, with fund managers or trustees executing the resulting reward (e.g., reduced cost of capital, continued credit payments) or mitigation pathways (e.g., remediation requirements, suspended disbursement)—giving investors continued assurance their capital is achieving verified outcomes, and giving verifiers an ongoing, paid role within the financial structure. This closes the cycle depicted in Figure 1, turning what would otherwise be a one-off transaction into an ongoing, self-correcting management system.

Because these actors rarely operate under one authority, the roadmap is best catalyzed by an intermediary organization—such as a landscape coordination body, conservation NGO, or regional cooperative (the value chain vs. the landscape portfolio)—that holds no direct stake in land-use outcomes and can therefore convene ecological actors, investors, jurisdictions, and communities who would not otherwise coordinate spontaneously. This reflects a common collective-action problem: because no single actor bears the full cost of coordinating the others, an independent broker is needed to get the cycle started, even as responsibility for sustaining alignment shifts toward the jurisdictional government’s role as convener (jurisdictional traceability) as the roadmap matures. Critically, this alignment does not rely on altruism: each actor’s continued participation is tied to a concrete benefit generated by the same mechanism that requires their involvement, from reduced due diligence costs to secured returns to recognized land rights.

## Conclusion

The development of sustainable production landscapes is not a utopian dream; it is a bio-economic necessity. Moving beyond unit-level certification requires landscape-scale stewardship that links ecological requirements and outcome verification with sustainable finance and integrated governance (Figure 1; Table 1)—a structure that, while developed here for tropical agro-commodity frontiers, is not conceptually specific to the tropics. Transcending the nature versus development dichotomy, future landscapes must link agro-commodity yields to the matrix’s biological health, rendering the traditional zero-sum game obsolete. By integrating ecological understanding and monitoring, sustainable finance, and jurisdictional traceability, we can ensure that the agro-commodity sector matures into a force for landscape restoration, securing both the global supply chains we depend on and the planetary biodiversity we cannot afford to lose.

**Table 1:** Overview of the discussed operational domains for biodiversity-positive production landscapes

Domain	Operational phase	Key mechanism/innovation	Output/verification
Ecology	system understanding, target setting and impact evaluation	field-based sensor grids (e.g., bioacoustics, camera traps, and drones)	field-validated bio-KPIs: non-manipulable data feeds for financial triggers
	response monitoring	before-after-control-impact (BACI) designs	attribution of biological gains to conservation interventions
Finance	asset structuring	landscape portfolios (bundling production and restoration)	landscape-linked asset class for institutional investors
	risk de-risking	blended finance (first-loss equity, concessional debt)	increased private capital flow to tropical frontiers
	programmatic incentives	smart contracts and performance-linked biodiversity credits	automated cost-of-capital reductions based on bio-KPIs
Governance	jurisdictional alignment	vertical policy coherence (zoning aligned with EUDR/KMGBF)	reduction in landscape-level leakage and legal certainty
	shared traceability	jurisdictional claims based on aggregated landscape data	regional low-risk verification for global markets
	social license	FPIC-based territorial contracts and smallholder cooperatives	long-term social sustainability and tenure security

## Acknowledgments

The author has no funding sources to declare.

## Declaration of interests

The author declares no competing interests.

## Declaration of generative AI and AI-assisted technologies in the writing process

During the preparation of this work, the author used Claude (Anthropic) to improve the language and readability of the manuscript. The author reviewed and edited the output as needed and takes full responsibility for the content of the published article.
